# Eurocode Shear Design of Coarse Recycled Aggregate Concrete: Reliability Analysis and Partial Factor Calibration

**DOI:** 10.3390/ma14154081

**Published:** 2021-07-22

**Authors:** João Pacheco, Jorge de Brito, Carlos Chastre, Luís Evangelista

**Affiliations:** 1 CERIS, IST, Universidade de Lisboa, 1049-001 Lisbon, Portugal; joaonpacheco@tecnico.ulisboa.pt; 2 CERIS, NOVA FCT, Universidade Nova de Lisboa, 2825-149 Caparica, Portugal; chastre@fct.unl.pt; 3 CERIS, ISEL, 1959-007 Lisbon, Portugal; luis.evangelista@isel.pt

**Keywords:** EN1992, prEN1992, shear, structural concrete, recycled aggregate concrete, reliability analysis, coarse recycled concrete aggregates, partial factor calibration

## Abstract

This paper contributes to the definition of design clauses for coarse recycled aggregate concrete. One of the main reasons for scepticism towards recycled aggregate concrete is the perceived notion that the heterogeneity of recycled aggregates may increase the uncertainty of the behaviour of concrete. Therefore, the paper uses structural reliability concepts to propose partial factors for recycled aggregate concrete’s design for shear failure. The paper builds upon a previous publication by the authors, in which the model uncertainty of recycled aggregate concrete elements designed for shear, with and without shear reinforcement, was compared with that of natural aggregate concrete elements. In that paper, the statistics of the model uncertainty for recycled aggregate concrete shear design were indeed found to be less favourable than those of natural aggregate concrete. Therefore, a partial factor for recycled aggregate concrete design is needed to ensure safety. This paper presents partial factors calibrated with explicit reliability analyses for different cases of design concerning beams (in the case of shear design of elements with shear reinforcement) and slabs (for the design of elements without shear reinforcement). For full incorporation of coarse recycled concrete aggregates and the design of elements without shear reinforcement, the calibrated partial factor reduces the design value of shear resistance by 10% (design with EN1992) or 15% (design with prEN1992) in comparison to natural aggregate concrete’s design. For the shear design of elements with shear reinforcement, the partial factor decreases resistance by 5% but a sensitivity analysis showed that the reduction might be, under pessimistic expectations, of up to 20%.

## 1. Introduction

### 1.1. Shear Resistance of Recycled Aggregate Concrete Elements

Research on the structural behaviour of recycled aggregate concrete (RAC) demonstrates the feasibility of using coarse recycled aggregates (RAs) for structural concrete applications [1,2]. This is relevant since RAs are produced from construction and demolition waste (CDW), whose recycling is a key objective of the European Union’s strategy for a circular economy [3]. Notwithstanding abundant research arguing in favour of RAC as a structural material [4,5], clear design guidelines are lacking. This is an obstacle for systematic applications of RAC, since without guidelines, designers, contractors and clients alike are reticent towards this structural material.

Guidelines for RAC are needed since RAs are different from coarse natural aggregates (NAs), changing the properties of concrete. The main differences between NAs and RAs are the following:

- NAs are composed of particles of a single type of stone (limestone, granite and basalt are the most common), while RAs are composed of a mix of several constituents (concrete, mortar, unbound stone, ceramics, glass, and other deleterious contaminants) of different quality and properties [6,7];
- This implies that, in general, RAs are weaker, more deformable, more porous and have larger water absorption than NAs [8,9];
- At the same time, the mechanical and durability properties of concrete are detrimentally affected by the incorporation of RAs: for the same compressive strength, RAC is typically found to have a smaller Young’s modulus, larger creep and shrinkage and worse durability properties [9,10,11]. Fracture energy and tensile strength are also detrimentally affected, especially when the strength class of concrete is larger [12,13];
- Regarding the structural behaviour of reinforced concrete, the use of RAs is found to result in larger short- and long-term deflections [14,15]. Ductility and resistance are only relevantly affected when the resistance mechanism relies relevantly on concrete rather than on reinforcement [1].

Research on RAC is mostly concerned with RAs produced from concrete waste, rather than RAs produced from mixed CDW, which includes concrete waste and the other aforementioned constituents. RAs produced from concrete waste are better suited for the production of RAC [16], since the other constituents (such as gypsum-based materials and weak ceramics) strongly impair the properties of concrete [6]. As such, this paper concerns the shear resistance of RAC elements made with coarse recycled concrete aggregates only.

Research on the shear resistance of RAC elements is comprehensive and includes the following topics:

- Comparison of the shear resistance of NAC and RAC elements with full incorporation of RAs (RAC100), with or without shear reinforcement [17,18,19,20];
- The influence of the incorporation ratio of RAs on shear resistance [21];
- The shear resistance of prestressed RAC beams [22];
- The shear resistance of RAC elements made with RAs that are treated with beneficiation methods [23];
- Meta-analyses that compare the shear resistance of NAC and RAC based on several investigations [24];
- The punching shear resistance of RAC elements [25,26,27].

The main findings of research on the shear resistance of RAC elements are that:

- The behavioural pattern of elements failing in shear is unaffected by the incorporation of RAs [17,20,21];
- The shear resistance of members without shear reinforcement decreases as the content of RAs increases [17,21,28,29];
- The previous finding is validated by a meta-analysis [24] that compares the model uncertainties ($\theta_{R}$) [30] of Eurocode shear resistance models for NAC and RAC design. These $\theta_{R}$ show that the resistance models of EN1992 [31] and prEN1992 [32] for elements without shear reinforcement overestimate the resistance of RAC in comparison to NAC elements;
- In most cases, the shear resistance of members with shear reinforcement is less affected by the incorporation of RAs [20,28].

The incorporation of RAs decreases shear resistance when shear reinforcement is absent because the RAs decrease the shear strength of concrete, as found in an appraisal [13] of several push-off experiments that compared the shear strength of NAC and RAC [33,34,35,36,37,38]. Shear strength decreases because:

- Aggregate interlock is a preponderant mechanism in shear strength mobilisation [39];
- RAs are weaker than NAs [8,40], i.e., RAs break more easily and limit aggregate interlock.

The decrease in shear strength of concrete caused by the use of RAs is followed by a decrease in shear resistance in the RAC element.

When elements have shear reinforcement, resistance models based on struts and ties are used in the design. In typical cases, these models are limited by the stress transfer of ties, so the only material property included in the resistance model is the reinforcement’s yield stress ($f_{y}$). This reflects the smaller relevance of concrete in the shear mechanism of common beams subjected to shear and implies that the detrimental effects of the incorporation of RAs on these cases of design is small.

Notwithstanding the comprehensive state-of-the-art knowledge on the shear behaviour of RAC elements, conventional research programmes cannot fully assess the need to change the provisions of NAC codes for RAC design. Research programmes that investigate the structural behaviour of reinforced concrete elements are carried out with a small number of specimens (typically a single specimen for each reinforcement layout and prototype geometry). Therefore, the influence of RAs on the structural reliability of reinforced concrete cannot be assessed. This is discussed in the next section.

### 1.2. Codified Shear Design of Recycled Aggregate Concrete

Since RAC is a viable structural material, research should be focused on giving designers the necessary means for the codified design of RAC structures. The ultimate limit state design of current codes has an underlying probabilistic basis. In the case of the Eurocodes, reinforced concrete design is made through characteristic values of material properties and load-effects, which underestimate the former and overestimate the latter. Moreover, partial factors are used to further decrease resistance and increase load-effects, providing an additional margin of safety. The following partial factors are typically used in the design equations of Eurocode reinforced concrete design:

- Partial factors $\gamma_{G}$ = 1.35 and $\gamma_{Q}=1.50$ increase actions. Permanent loads are multiplied by $\gamma_{G}$ and variable loads are multiplied by $\gamma_{Q}$;
- Partial factors $\gamma_{S}$ = 1.15 and $\gamma_{C}=1.50$ decrease material properties or resistance (depending on the resistance model). In most cases, the characteristic yield stress of the reinforcement is divided by $\gamma_{S}$, while the compressive strength of concrete is divided by $\gamma_{C}$;
- $\gamma_{C}$ may be modelled as $\gamma_{C}=\gamma_{c}\times \gamma_{Rd}$, where $\gamma_{c}$ is a partial factor for material variability and $\gamma_{Rd}$ is a partial factor that accounts for the uncertainty in geometry and in resistance modelling.

The margins of safety provided by the partial factors of the Eurocodes are calibrated based on the variability involved in structural design and require that the safety of structural design complies with the expectations of society. Any new guideline for the ultimate limit state design of RAC (the case of this paper for shear design) needs to take this into account. Therefore, the paper calibrates a partial factor for shear design ($\gamma_{RAC}$).

Codes have a probabilistic basis because most of the variables that are relevant to structural design are subjected to uncertainty. Therefore, the calibration of codes requires that load-effects, geometry, material properties and the parameters related to modelling be modelled as stochastic variables. Then, reliability analyses are used to calibrate factors used in the design equations of codes. The calibration of these factors ensures that the reliability index (β) of codified design complies with target values based on the expectation of society regarding safety.

Since RAs are more heterogeneous [41] and weaker than NAs:

- The behaviour of RAC may be more variable than that of NAC;
- The resistance models used for NAC may not be as representative for RAC.

The latter statement is justified by the different stress paths, crack propagation and damage progression of RAC in comparison to NAC [42,43]. This influences the $\theta_{R}$ of the resistance models of codes. Differences in $\theta_{R}$ are relevant in the context of structural reliability, since they are accounted for in the definition of the partial factors of the Eurocodes [44]. If these uncertainties are affected by the incorporation of RAs, then the assumptions and partial factors of EN1992 [31] may be unsafe for RAC design.

In a recent publication [24], the authors proposed a preliminary partial factor $\gamma_{RAC}$ that accounts for the $\theta_{R}$ of RAC in the safety of the shear design of elements without shear reinforcement when either EN1992 [31] or prEN1992 [32] is used. This preliminary partial factor $\gamma_{RAC}$ was put forward based on a methodology that conforms with Annex C of ISO2394 [45] and Annex D of EN1990 [44].

A database of validated experiments on the shear resistance of NAC and RAC was gathered and stochastic models for the model uncertainty ($\theta_{R}$) of the shear resistance modelling of EN1992 [31] and prEN1992 [32] were estimated for NAC and RAC. Afterwards, these $\theta_{R}$ were used to define the preliminary partial factor $\gamma_{RAC}$ for the shear design of elements without shear reinforcement. Due to specificities of the shear resistance models of EN1992 [31] and prEN1992 [32], partial factors for NAC design ($\gamma_{NAC}$) were also proposed.

However, the methodology of Annex C of ISO2394 [45] and Annex D of EN1990 [44] is based on assumptions that may not be accurate for the reliability of elements designed for shear. This occurs because shear resistance models have higher scatter than most other resistance models [46] and assumptions concerning the direction cosines (α) presented in ISO2394 [45] and EN1990 [44] may not be accurate. Therefore, this paper removes these assumptions from the estimates of $\gamma_{NAC}$ and $\gamma_{RAC}$ for elements without shear reinforcement through calibration based on reliability analysis.

Concerning elements with shear reinforcement, no partial factor is proposed in [24] since research on RAC shear resistance of this type of element is not sufficient for a sound database. Thus, reliability methods based on sensitivity analyses are used to propose $\gamma_{RAC}$.

### 1.3. Objectives

The main contribution of this paper is the proposal of design guidelines for the shear design of RAC elements with and without shear reinforcement. These guidelines are based on the Eurocode format and include a partial factor $\gamma_{RAC}$ for RAC design when either EN1992 [31] or prEN1992 [32] is used. The latter document presents significant changes [47] in comparison with the shear strength model of the current version of EN1992 [31]. The paper has the following objectives:

- Proposal of a resistance format with a specific partial factor for RAC design;
- Reliability analyses for representative cases of design using the stochastic models for $\theta_{R}$ proposed in [24];
- Calibration of $\gamma_{RAC}$ to be used in the resistance format proposed;
- Sensitivity analyses to understand the robustness of the calibrated $\gamma_{RAC}$ for the shear design of elements with shear reinforcement.

This paper is part of an effort by the authors towards the reliability-based structural design of RAC. So far, publications have addressed:

- The within-batch variability of the mechanical properties of RAC [48];
- A probabilistic factor for the conversion of the compressive strength tested in 150 mm cubes to the compressive strength used in design codes (ϕ 150 mm × 300 mm cylinders) [49];
- The scatter of the compressive strength vs. tensile strength and compressive strength vs. Young’s modulus relationships of EN1992 [31], Model Code 2010 [50] and prEN1992 [51] and ACI318 [52] when used for RAC [12];
- The model uncertainty of the flexural resistance models of EN1992 [31], prEN1992 [51], and ACI318 [52] for RAC [4];
- The model uncertainty of the shear resistance models of EN1992 [31], prEN1992 [51], and Model Code 2010 [53] for RAC [24];
- The model uncertainty of the bond strength and the reliability-based calibration of the development length of ribbed steel reinforcement for RAC when fib Bulletin 72 or prEN1992 [51] is used [40];
- The reliability-based calibration of concrete cover for EN1992 [31] design of RAC for chloride-prone environments [54].

## 2. Design Equations of the Eurocode Format

### 2.1. Design of Members without Shear Reinforcement

According to EN1992 [31], the design value for the shear resistance of elements without shear reinforcement is:

(1) $$V_{Rd}=\left(\frac{0.18}{\gamma_{C}}\;\times \;k\;\times \;\sqrt[{3}]{100\;\rho_{l}\;f_{ck}}\right)\;\times \;d\;\times \;b\;\geq \;\nu_{min}\;\times \;d\;\times \;b$$

where:

- $\gamma_{C}$ is the partial factor for concrete;
- b is the width of the web of the beam;
- d is the effective depth of the beam;
- $k=1+\sqrt{\frac{200}{d}}\leq 2.0$ accounts for size effects. In this equation, d is in mm;
- $\rho_{l}$ is the geometric ratio of the longitudinal tensile reinforcement. In this equation, $\rho_{l}\leq 2\%$;
- $f_{ck}$ is the characteristic compressive strength of concrete and is in MPa;
- $\nu_{min}=0.035\times k^{3/2}\times \sqrt[{2}]{\;f_{ck}}$ is the minimum shear stress. This condition is always complied with in this paper and is not mentioned from here on.

Equation (1) uncouples $\gamma_{C}$ from $f_{ck}$. This is not a common option of the resistance models of EN1992 [31] but allows that:

- In the case of NAC, $\gamma_{C}$ is replaced with $\gamma_{NAC}$;
- In the case of RAC, $\gamma_{C}$ is replaced with $\gamma_{RAC}$.

Partial factor $\gamma_{NAC}$ differs from 1.50, the value of $\gamma_{C}$ of EN1992 [31], because of the preliminary findings of [24]. These findings suggest that using $\gamma_{C}=1.50$ may lead to overly safe (thus uneconomical) design in the case of the shear design of elements without reinforcement. If the reliability analysis developed in this paper finds that β is below expectations, $\gamma_{NAC}$ will be calibrated. $\gamma_{RAC}$ is calibrated using reliability analyses on representative cases of design. The calibration criterion is stated in Section 3.1.

In the case of prEN1992 [32], the design value for the shear resistance of elements without shear reinforcement is:

(2) $$V_{Rd}=\left(\frac{0.6}{\gamma_{C}}\times \sqrt[{3}]{100\rho_{l}\times f_{ck}\times \frac{d_{dg}}{a_{v}}}\right)\;\times \;d\;\times \;b\;\geq \;V_{Rd,c,min}$$

where:

- $d_{dg}=16+d_{max}\leq 40\;\mathrm{mm}$ if $f_{c}\leq 60\;\mathrm{MPa};$
- $d_{dg}=16+{\left(d_{max}/f_{c}\right)}^{2}\leq 40\;\mathrm{mm}$ if $f_{c}>60\;\mathrm{MPa}$;
- $a_{v}=\sqrt{\frac{a_{cs}}{4}\times d}$, where $a_{cs}=\left|M_{cs}/V_{cs}\right|\left(\geq d\right)$;
- $a_{v}=d$ if $a_{cs}>4d$;
- $V_{R,c,min}=\left(\frac{10}{\gamma_{C}}\right)\times \sqrt[{2}]{\frac{f_{ck}}{f_{yk}/\gamma_{s}}\times \frac{d_{dg}}{d}}$;
- $f_{yk}$ is the characteristic yielding stress of the longitudinal reinforcement;
- $\gamma_{s}=1.15$ is the partial factor of steel reinforcement;
- $M_{cs}$ and $V_{cs}$ are the bending moment and shear stress at the control section;
- In this code, no limit on the $\rho_{l}$ of EC2 (2004) is imposed.

Alternatively, this code allows Equation (2) to be replaced with a simplified and conservative expression. This paper calibrates partial factors for Equation (2) only, but these factors may be used in both expressions. The same option is taken in prEN1992 [32], where a single $\gamma_{C}$ is used for NAC.

As for the design equation of EN1992 [31], partial factor $\gamma_{C}$ is replaced with $\gamma_{NAC}$.

### 2.2. Design of Members with Shear Reinforcement

EN1992 [31] and pEN1992 [32] use the same resistance model for the design value of the resistance of elements with shear reinforcement. This resistance model is based on struts and ties. The load-bearing capacity of ties is given by:

(3) $$V_{Rd,tie}=\frac{A_{sw}}{s}\times 0.9d\times \frac{f_{yk}}{\gamma_{S}}\times \cot\left(\Omega \right)\;\leq \;V_{R,strut}$$

where:

- $A_{sw}$ is the shear reinforcement area;
- s is the distance between shear reinforcement;
- $f_{yk}$ is the characteristic yield stress of the shear reinforcement;
- $\gamma_{S}=1.15$ is the partial factor for reinforcement strength, including geometric and modelling uncertainty;
- Ω is the angle of the strut with the longitudinal axis of the element. This angle may be assumed as any value in the region of 21.8 to 45°.

The model assumes that the resistance of the compression struts is:

(4) $$V_{Rd,strut}=\frac{b\times 0.9d}{\cot\left(\Omega \right)\;+\;\tan\left(\Omega \right)}\times \frac{\nu_{1}\times f_{ck}}{\gamma_{C}}.$$

EN1992 [31] defines $\nu_{1}=0.6$ if $f_{ck}<60\;\mathrm{MPa}$, while for a design with pEN1992 [32], $\nu_{1}=0.5$ may be used.

EN1992 [31] and pEN1992 [32] allow designers to assume a value for angle Ω. In this paper, the most common option of designers was used: Ω was assumed as 30°.

In the typical cases found in conventional reinforced concrete design, $V_{Rd,tie}$ limits the resistance mechanism. Hereafter, all mentions in the paper to $V_{Rd}$ concern shear strength design limited by the design value of resistance of ties (Equation (3)).

Equation (3) does not include $\gamma_{C}$, since its underlying resistance model does not consider the influence of concrete on the resistance mechanism. Nevertheless, concrete bears part of the shear force (this is acknowledged in refined formulae, such as the shear resistance model of Model Code 2010 for Level of Approximation 3 [53]) and the incorporation of RAs will affect the resistance mechanism. Therefore, a partial factor for RAC is calibrated.

## 3. Reliability Analysis for Partial Factor Calibration

### 3.1. Calibration Procedure and Reliability Method

Equations (1)–(3) result in five expressions for the design value of resistance that need to be calibrated. Equations (5)–(8) concern the design of elements without shear reinforcement and are adaptations of either Equation (1) for EN1992 [31], or Equation (2) for prEN1992 [32]. Equation (9) is intended for the shear design of RAC elements with shear reinforcement. In this case, EN1992 [31] and pEN1992 [32] use the same resistance model and design equation.

(5) $$V_{Rd,EN1992,NAC}=\left(\frac{0.18}{\gamma_{NAC}}\times k\times \sqrt[{3}]{100\;\rho_{l}\times f_{ck}}\right)\;\times \;d\;\times \;b,$$

(6) $$V_{Rd,EN1992,RAC}=\left(\frac{0.18}{\gamma_{RAC}}\times k\times \sqrt[{3}]{100\;\rho_{l}\times f_{ck}}\right)\;\times \;d\;\times \;b,$$

(7) $$V_{Rd,prEN1992,NAC}=\left(\frac{0.6}{\gamma_{NAC}}\times \sqrt[{3}]{100\rho_{l}\times f_{ck}\times \frac{d_{dg}}{a_{v}}}\right)\;\times \;d\;\times \;b,$$

(8) $$V_{Rd,prEN1992,RAC}=\left(\frac{0.6}{\gamma_{RAC}}\times \sqrt[{3}]{100\rho_{l}\times f_{ck}\times \frac{d_{dg}}{a_{v}}}\right)\;\times \;d\;\times \;b,$$

(9) $$V_{Rd,RAC}=\frac{1}{\gamma_{RAC}}\times \left[\frac{A_{sw}}{s}\times 0.9d\times \frac{f_{yk}}{\gamma_{S}}\times \cot\left(\Omega \right)\right].$$

The calibration concerns partial factors $\gamma_{NAC}$ and $\gamma_{RAC}$ only. All other partial factors (see Section 1.2) are kept as those of the Eurocodes, so that the design guidelines proposed in this paper are easily implemented. The calibration is made for concrete with 50% incorporation of RAs (RAC50) and for concrete with full incorporation of RAs (RAC100), but partial factors for intermediate incorporation ratios of RAs may be linearly interpolated.

In addition, to ensure an unbiased estimate of β, the reliability analyses are made for the exact correspondence of the design value of load-effects ($E_{d}$) with the design value of resistance ($V_{Rd}$).

The load combination presented in Equation (6.10) of EN1990 [44] is used to calculate $E_{d}$. A permanent load and a single variable load are assumed:

(10) $$E_{d}=\gamma_{G}\times P_{k}+\gamma_{Q}\times Q_{k}$$

where $\gamma_{G}=1.35$ is the partial factor for permanent loads and $\gamma_{Q}=1.50$ is the partial factor for variable loads, both including modelling uncertainty; $P_{k}$ is the characteristic value of permanent loads; and $Q_{k}$ is the characteristic value of the variable load.

The calibration of these expressions is made through reliability analyses using an iterative procedure. For each of Equations (5)–(9):

1. Cases of design are defined;
2. The load combination presented in Equation (10) is used to determine $E_{d}$;
3. An iterative process takes place, beginning with the preliminary proposal of the authors [24] for $\gamma_{NAC}$ and $\gamma_{RAC}$. For a given $E_{d}$, the equation for the design value of resistance is used to design the structural element;
4. A reliability analysis takes place, in which a limit state function of the type $g_{x}=R-E$ is used to determine β. R is the random outcome of resistance and E is the random outcome of load-effects. The limit state functions used are presented in Section 3.2 and Section 3.3;
5. When β is below expectations, a new partial factor is checked and a new iteration (starting at step 3 of this bullet list) takes place;
  - The calibration criteria for shear design of elements without shear reinforcement are that:
    - In the case of NAC, $\gamma_{NAC}$ ensures that the target reliability index ($\beta_{target}$ = 3.8, since reliability class 2 and a 50-year reference period are considered [44]) is complied with in the majority of cases of design. Moreover, the β value should be similar to those obtained in seminal reliability assessments of the Eurocodes [55,56];
    - In the case of RAC, the criterion is that $\gamma_{RAC}$ results in a similar *β* value to that obtained when $\gamma_{NAC}$ is used for NAC design;
  - In the case of the shear design of elements with shear reinforcement, no partial factor for NAC is used and the criterion is that the calibrated $\gamma_{RAC}$ leads to a similar β value to that when NAC elements are designed.

The reliability analyses use a Rosenblatt transform to convert stochastic variables to the standard normal space and the reliability algorithm used is a First Order Reliability Method [57]. The discussion of the results of the reliability analyses (Section 5) is mostly focused on *β* and on $\alpha^{2}$, the square of the direction cosines of the stochastic variables. Since all stochastic variables are independent and uncorrelated, the $\alpha^{2}$ of a stochastic variable is the percentage of the total uncertainty that is due to the uncertainty in that stochastic variable [58].

To ensure that the partial factors are representative of common design, the reliability analyses concern cases of design that are common for either resistance mechanism. Therefore:

- The partial factors for shear resistance of elements without shear reinforcement are calibrated for the shear design of slabs;
- The partial factor for shear resistance of elements with shear reinforcement is calibrated for the shear design of beams.

The cases of design are presented in Section 4.1.

### 3.2. Limit State Function for Slabs without Shear Reinforcement

The limit state functions compare the random outcome of resistance and load-effects. Thus, all parameters of design that are subjected to uncertainty are modelled as stochastic variables. These include the magnitude and modelling of loads, material and geometric uncertainty, and $\theta_{R}.$ Equations (11) and (12) are the limit state functions used for the reliability analysis of design using EN1992 [31] and prEN1992 [32] for RAC. In these expressions, parameters that include stochastic modelling are presented in bold.

(11) $$g{\left(x\right)}_{,EN1992}=\theta_{R}\;\times \;\left[\left(0.18\times k\times \sqrt[{3}]{100\;\rho_{Asl}\times f_{c}}\right)\;\times \;d\;\times \;b\right]\;-\;\theta_{E}\;\times \;\left(P+Q\right)$$

where $k=1+\sqrt{\frac{200}{d}}\leq 2$ and $\rho_{Asl}=\frac{A_{s}}{\;d\times b}$.

(12) $$g{\left(x\right)}_{,prEN1992}=\theta_{R}\;\times \;\left[\left(0.6\times \sqrt[{3}]{100\rho_{Asl}\times f_{c}\cdot \frac{d_{dg}}{a_{v}}}\right)\;\times \;d\;\times \;b\right]\;-\;\theta_{E}\;\times \;\left(P+Q\right).$$

$\theta_{E}$ is the model uncertainty of load-effect modelling, $f_{c}$ is the random outcome of compressive strength, and P and Q are the random outcomes of permanent and variable loading.

The stochastic models are presented in Section 4.2.

### 3.3. Limit State Function for Beams with Shear Reinforcement

In the case of elements with shear reinforcement, the same equation is used for EN1992 [31] and prEN1992 [32]. This $g\left(x\right)$ is meant for reliability analyses when the design is made with Equation (3) for NAC or Equation (9) for RAC.

(13) $$g\left(x\right)=\theta_{R}\;\times \;\left(\frac{A_{sw}}{s}\times 0.9d\times f_{y}\times \cot\left(\Omega \right)\right)\;-\;\theta_{E}\;\times \;\left(P+Q\right).$$

## 4. Cases of Design and Modelling

### 4.1. Cases of Design

Since the results of reliability analyses depend on the specific cases of design [59], several cases of design covering conventional reinforced concrete design were used in the calibration procedure.

**Figure 1:**
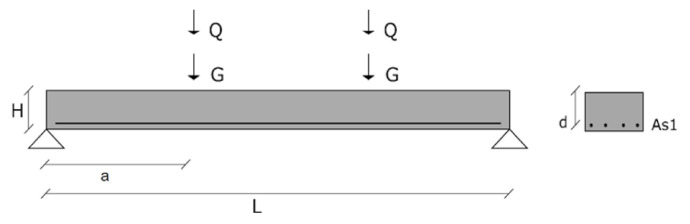
Case of design of the reliability analysis. Slab without shear reinforcement.

In the case of slabs, the cases of the design were based on Figure 1 and included different cross-sectional height and longitudinal reinforcement detailing. Two compressive strength classes were analysed: C25/30 and C40/50 [60]. RAC applications of higher strength classes than C40/50 are not expected in the near future, due to the reservations of construction agents and because of the restrictions of standards [32] and national specifications [61].

Table 1 presents the main parameters of design of the five slabs analysed when prEN1992 [32] was used. In this table, $\Phi_{Asl}$ is the diameter of longitudinal reinforcement, s is its spacing, H is the height of the beam and cy is the concrete cover in the vertical direction. Despite the reliability analyses concerning three incorporation ratios of RAs (NAC, RAC50, and RAC100), only NAC slabs are presented, since the design of RAC depends on the iterative calibration of $\gamma_{RAC}$.

The $\gamma_{NAC}$ and $\gamma_{RAC}$ used in the first iteration of the calibration procedure are presented in Table 2 and were derived in [24].

Table 3 shows the NAC cases when the design was made with EN1992 [31]. In these cases, the slabs were thicker than those of prEN1992 [32] to prevent parameter k from being limited to 2.0—see Equation (1). The parameters of design were adjusted so that the $V_{Rd}$ values of both prEN1992 [32] and EN1992 [31] were the same.

In the case of beams, a single case of design was studied because:

- The uncertainty in the outcome of resistance of this type of design is virtually lognormally distributed and reliability is predominantly dependent on the moments of $\theta_{R}$ and $f_{y}$, which do not depend on the case of design. This occurs because Equation (13) has a multiplicative nature, is mainly composed of lognormal distributions, and depends mostly on $\theta_{R}$ and $f_{y}$;
- The uncertainty in the outcome of load-effects depends on $\theta_{E}$, P, and Q only. Since the statistics of $\theta_{E}$ are fixed for all cases of design, loads are given by P+Q and the variability of P and Q are defined in terms of their coefficient of variation (CoV), different cases of design lead to similar uncertainty in the outcome of load-effects.

Thus, in the case of beams, different cases of design result in the same outputs of reliability analysis, including β as long as $V_{Rd}$ is equal to the design value of load-effects (which is already a condition of the reliability analyses performed). Table 4 presents the case of design analysed. As in the case of Table 1, only NAC is presented.

The design complied with the maximum spacing between shear reinforcement and with the minimum geometric ratio of shear reinforcement ($\rho_{w}$) of either code [31,32]. As stated in Section 2.2, $V_{Rd}$ is given by $V_{Rd,tie}$ in all cases. $\gamma_{RAC}=1.0$ in the first iteration of the calibration stage.

Each case of design of both beams and slabs included seven separate reliability analyses, since different ratios of variable to total load were analysed. For that purpose, parameter χ was defined as shown in Equation (14). The analysis of χ vs. *β* relationships was made because other code calibration procedures have shown that the β  values of Eurocode design depend on χ [55,56].

(14) $$\chi =\frac{\gamma_{G}\times G_{k}}{\gamma_{G}\times P_{k}+\gamma_{Q}\times Q_{k}}.$$

### 4.2. Deterministic and Stochastic Modelling

The deterministic and stochastic modelling is shown in Table 5, Table 6 and Table 7. These models are needed for the design equations, which were presented in Equations (3) and (5)–(9), and limit state functions, presented in Equations (11)–(13).

The incorporation of RAs does not affect reinforcement, loads, load-effect modelling, or geometry; therefore, the criterion was to use the stochastic models of seminal publications (those of the Joint Committee for Structural Safety [62] and of other relevant documents—e.g., concerning the calibration and partial factor assessment of the Eurocodes). Table 5 shows the references of each stochastic model. In the case of the compressive strength of concrete, stochastic modelling was made after the proposal of Bartlett and McGregor [63]. Parameter λ of this proposal was defined based on the quality control records of Portuguese ready-mixed concrete production for the year 2017 [64].

No modelling for b (the width of the web of the cross-section) is presented in Table 5 since this parameter is used for the shear resistance of elements without shear—see Equations (5)–(8)—but the cases of design concerned slabs (and geometric uncertainty in this case was negligible). In addition, no modelling for variability of height is presented since vertical deviations concerned the effective depth (given by the effective depth, which accounts for the uncertainty in cover).

The $\theta_{R}$ of the elements without shear reinforcement is presented in Table 6 and its derivation may be consulted in [24]. No deterministic model is presented for $\theta_{R}$ because model uncertainties were omitted from design equations—see Equations (3) and (5)–(9).

Since research on the shear resistance of elements with shear reinforcement that comply with the criteria typically used in the assessment of $\theta_{R}$ [78] is not abundant, the stochastic models presented in [24] for the design of elements with shear reinforcement are preliminary, and additional research is needed prior to the definition of statistics for $\theta_{R}$. Therefore, the approach taken in this paper was to perform a sensitivity analysis for two assumptions:

- Assumption 1, in which the statistics of NAC are those presented in *fib* Bulletin 80 [79] for $\rho_{w}$ ∙$f_{yd}$ between 1 and 2 MPa. Concerning RAC, this case assumes that the mean value of $\theta_{R}$ is unaffected by the incorporation of RAs, but the standard deviation increases as the RA incorporation ratio increases;
- Assumption 2, in which the statistics of NAC are those presented in *fib* Bulletin 80 [79] for $\rho_{w}$ ∙$f_{yd}$ between 1 and 2 MPa and pessimistic expectations for the influence of RAs on the mean value and standard deviation of $\theta_{R}$ are assumed.

Table 7 summarises these assumptions. The statistics of this $\theta_{R}$ were characterized by high CoV for both NAC and RAC. This was expected to lead to small *β* values.

## 5. Results

### 5.1. Slabs without Shear Reinforcement

#### 5.1.1. Design with prEN1992

**Figure 2:**
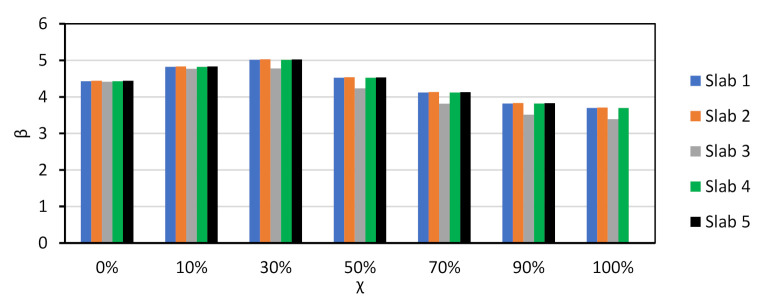
*χ* vs. β for shear resistance design of NAC slabs without shear reinforcement. prEN1992 [32]. $\gamma_{NAC}=1.40$.

The results of the reliability analyses are presented in Figure 2 for all NAC slabs. As shown there, the β values of the slabs are similar. The same β vs. χ trend reported in other documents concerning Eurocode design [55,56] was observed. Moreover, the 50-year β that resulted from a design with $\gamma_{NAC}=1.40$ was an analogue to that of Eurocode reliability assessments and calibration efforts [67,68,73], and no calibration of $\gamma_{NAC}$ was needed.

**Figure 3:**
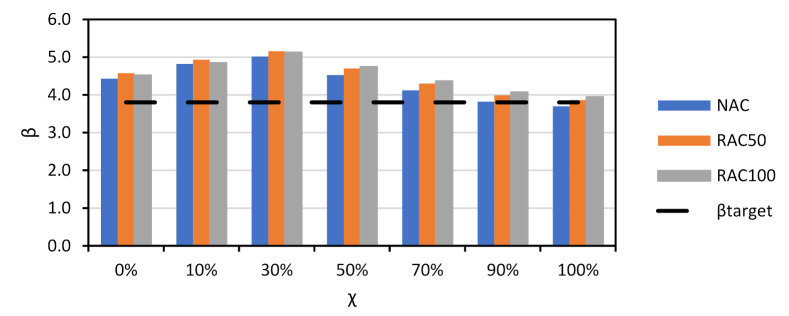
*β* vs. *χ* of Slab 1 for NAC and RAC. prEN1992 [32]. $\gamma_{RAC}$ of Table 2 (not calibrated).

Since the trends observed for all slabs were similar, the discussion of results is focused on Slab 1. Figure 3 compares the β vs. χ relationship of NAC, RAC50 and RAC100. It is shown that: (i) β was above $\beta_{target}=3.80$ in all cases except for NAC and χ=100%; (ii) the β of RAC was above that of NAC, which means that the partial factor $\gamma_{RAC}$ presented in Table 2 for RAC50 and RAC100 may be decreased.

**Figure 4:**
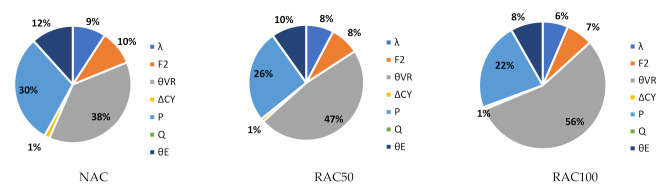
$\alpha^{2}$ for χ=0 and Slab 1 and the preliminary partial factors for shear design of elements without stirrups presented in Part 3. prEN1992 [32]. $\gamma_{NAC}=1.40$; $\gamma_{C,V,RAC50}=1.60;\;\gamma_{C,V,RAC50}=1.70$.

To better understand the relative importance of all sources of uncertainty, Figure 4 shows the $\alpha^{2}$ values of Slab 1 for χ=0 and NAC, RAC50, and RAC100 design. The figure shows that the uncertainties in $\theta_{R}$ and P are the most relevant for the overall uncertainty. Moreover, as the incorporation ratio of RA increased, the $\alpha^{2}$ of $\theta_{R}$ increased. This was due to the detrimental influence of RAs on the statistics of $\theta_{R}$ (see Table 6) and suggested that, notwithstanding the preliminary partial factor $\gamma_{RAC}$ leading to a larger β value than that of NAC, a specific partial factor for $\gamma_{RAC}$ is still needed.

Findings for other χ values were similar to those reported in Figure 3. The only noteworthy differences are that as χ increased: (i) the $\alpha^{2}$ of Q increased; (ii) the $\alpha^{2}$ of P decreased relevantly; and (iii) the $\alpha^{2}$ of all other stochastic variables decreased proportionally to the $\alpha^{2}$ reported in Figure 3.

**Figure 5:**
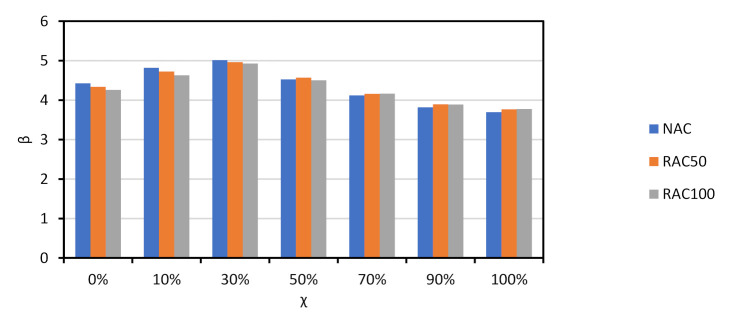
*β* vs. *χ* of Slab 1 for NAC and RAC. prEN1992 [32]. $\gamma_{NAC}=1.40$ and $\gamma_{CRAC50}=1.50,\;\gamma_{RAC100}=1.60$ (after calibration).

The calibration procedure presented in Section 3.1 was used. Figure 5 and Table 8 show that the β value of the RAC design became similar to that of NAC when $\gamma_{RAC50}=1.50$ and $\gamma_{RAC100}=1.60$.

#### 5.1.2. Design with EN1992

**Figure 6:**
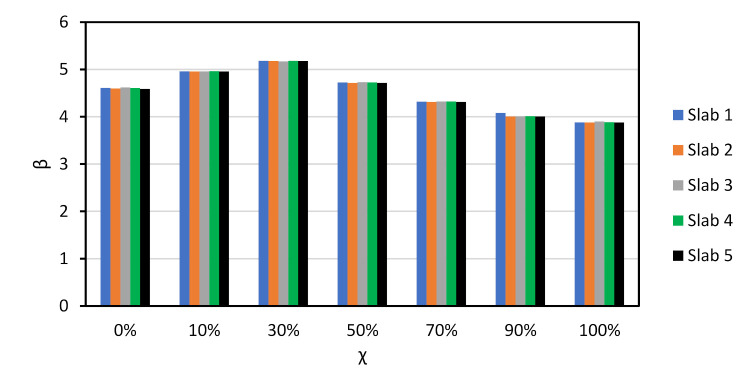
*χ* vs. β for shear resistance design of NAC slabs without shear reinforcement. EN1992 [31]. $\gamma_{NAC}=1.45$; $\gamma_{RAC50}=1.55;\;\gamma_{RAC100}=1.60$ (no calibration needed).

Figure 6 presents the relationship between β and χ of all NAC slabs designed with EN1992 [31]. As in the case of prEN1992 [32], the results of all slabs were similar. Moreover, the comparison between Figure 4 and Figure 6 showed that the design with either code resulted in similar β values, notwithstanding the smaller value $\gamma_{NAC}$ and the smaller mean $\theta_{R}$ of the resistance model of prEN1992 [32] in comparison to those of EN1992 [31]. This means that shear design of NAC members without shear reinforcement tends to be more economical when prEN1992 [32] is used, due to the better precision of its resistance model.

The reliability analyses also found that the preliminary partial factors proposed in [24] and presented in Table 2 resulted in *β* values that were similar to those of the NAC design. Table 9 shows these results.

**Figure 7:**
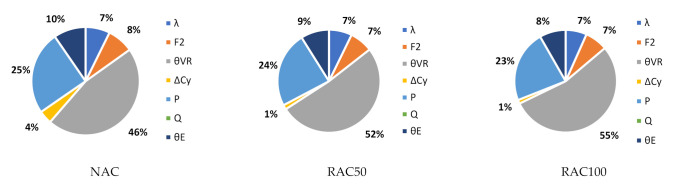
$\alpha^{2}$ for χ=0 and Slab 1. Slabs without shear reinforcement. EN1992 [31]. $\gamma_{NAC}=1.45$ and $\gamma_{CRAC50}=1.55,\;\gamma_{RAC100}=1.60$ (no calibration needed).

Figure 7 is an example (Slab 1 and *χ* = 0) of the $\alpha^{2}$ of all stochastic variables. It shows that the trends reported for designs with prEN1992 [32] were also observed for EN1992 [31].

### 5.2. Beams with Shear Reinforcement

**Figure 8:**
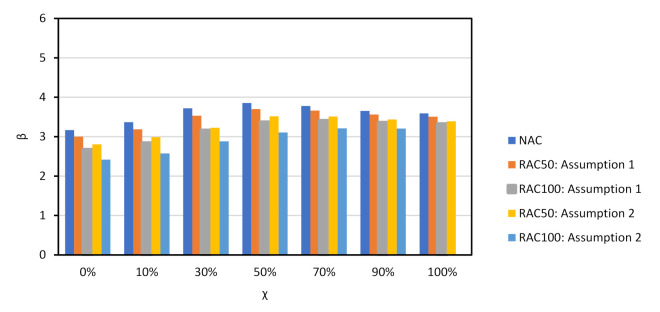
*χ* vs. β for shear resistance design of NAC and RAQ beams with shear reinforcement. EN1992 [31] and prEN1992 [32]. $\gamma_{RAC}=1.0$ (not calibrated).

Figure 8 shows the β vs. χ relationship of NAC and RAC beams. Partial factor $\gamma_{RAC}$ is yet to be calibrated ($\gamma_{RAC}=1.0$ at this stage). As expected, the incorporation of RAs resulted in a relevant decrease of reliability, particularly when Assumption 2 for $\theta_{R}$ (Table 7) was analysed. Moreover, the β value achieved for the shear design of elements with shear reinforcement was below $\beta_{target}$ (even in the case of NAC); for *χ* it was in the region of 0 to 10% and for *χ* above 70%, which are uncommon cases of reinforced concrete design [55].

**Figure 9:**
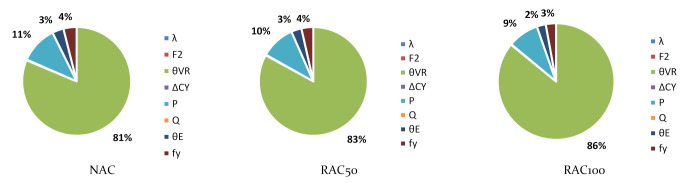
$\alpha^{2}$ for χ=0 and $\theta_{R}$ Assumption 1. EN1992 [31] and prEN1992 [32]. $\gamma_{RAC}=1.0$ (not calibrated).

Figure 9 shows large values of the $\alpha^{2}$ of $\theta_{R}$, which demonstrated that $\theta_{R}$ was the cause of most of the uncertainty in this type of design. Figure 9 only concerns Assumption 1 of Table 7 since findings for Assumption 2 followed the same rationale.

**Figure 10:**
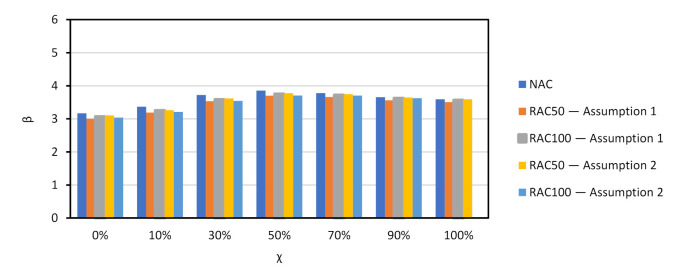
*β* vs. *χ* for both assumptions for $\theta_{R}$. EN1992 [31] and prEN1992 [32]. Results of the calibration: Assumption 1: $\gamma_{RAC50}=1.00;$ $\gamma_{RAC100}=1.05$; Assumption 2: $\gamma_{RAC50}=1.10;$ $\gamma_{RAC100}=1.20$ (after calibration).

Partial factor $\gamma_{RAC}$ was calibrated and Figure 10 shows the results of calibration. As observed, the β value of the RAC design was equivalent to that of the NAC design when the calibrated partial factors were used. The next section presents all calibrated partial factors.

Since the $\theta_{R}$ for elements with shear reinforcement was based on scarce data and assumptions, the elasticities of the mean and of the standard deviation [58] of this stochastic variable are studied and presented in Table 10.

These elasticities showed that, if the statistics of the $\theta_{R}$ for elements with shear reinforcement differed from those assumed, the influence on *β* would be relevant. For instance:

- For Assumption 1 and *χ* = 50%, the beam made with RAC100 has a 50-year β of 3.75. Assumption 1 models $\theta_{R}$ with a mean of 1.25;
- If the mean of $\theta_{R}$ is 1.20 instead of 1.25, the actual 50-year β would correspond to roughly:

(15) $$\beta =3.75\;\cdot \;\left[1+\left(\frac{1.20-1.25}{1.25}\right)\;\times \;1.99\right]=3.45.$$

This corresponds to a decrease in *β* of 8%. This decrease emphasized that further experiments on the shear resistance of beams with stirrups should be performed prior to definite proposals for $\theta_{R}$ and partial factors for RAC design in order to base the statistics of this $\theta_{R}$ on a comprehensive set of data.

### 5.3. Recommendations for Design

Partial factors for shear design of elements with and without shear reinforcement were calibrated for the design of:

- Elements without shear reinforcement using EN1992 [31]—Equations (5) and (6);
- Elements without shear reinforcement using prEN1992 [32]—Equations (7) and (8);
- Elements with shear reinforcement using either EN1992 [31] or prEN1992 [32]—Equations (3) and (9).

The recommended partial factors, which were discussed in Section 5.1 and Section 5.2, are presented in Table 11.

These partial factors ensure that the β values of reinforced concrete elements made with incorporation of RAs are similar to those of conventional reinforced elements designed for analogue conditions. Partial factors for other incorporation ratios may be determined by linear interpolation.

The partial factor calibrated for Assumption 2 of the shear design of elements with shear reinforcement is a conservative upper bound of the implications of RAs for shear design, and its main purpose is to show that additional experiments on the shear resistance of elements with shear reinforcement are needed prior to a definite calibration of a partial factor for this resistance model.

## 6. Conclusions

This paper provided partial factors for the shear design of reinforced concrete elements made with the incorporation of coarse recycled aggregates produced from concrete waste. The paper addressed the design of elements with and without shear reinforcement. The design equations concerned two codes: the current version of EN1992 and prEN1992 (the next generation of EN1992, under approval by CEN).

An overview of research on the shear resistance of recycled aggregate concrete elements was provided. The fundamental reason for recycled aggregates affecting the uncertainty in shear resistance modelling was stated: since recycled aggregates are weaker than natural aggregates, aggregate interlock decreases without this being accounted for by the shear resistance model. The probabilistic basis of structural codes was discussed and the development of design guidelines for Eurocode recycled aggregate concrete design was contextualised with the partial factor format of the Eurocodes.

A partial factor was then added to the design equations of EN1992 and prEN1992 and calibrated using a procedure that follows the general rules and recommendations of ISO2394 and EN1990. Relevant increases in the partial factor were found for the design of elements without shear reinforcement and, without the calibrated partial factor for recycled aggregate concrete design, structural safety was compromised. In the case of elements with shear reinforcement, since research is not as comprehensive, the calibrated partial factor was preliminary and defined based on a sensitivity analysis and assumptions. It was found that the partial factor was sensitive to deviations in the statistics of the model uncertainty and that additional research is recommended prior to the definite calibration of a partial factor. In the meantime, a partial factor calibrated based on engineering judgement and either moderate or fairly pessimistic assumptions was calibrated and proposed.

The authors recommend future research on the punching shear resistance of recycled aggregate concrete; research and partial factor calibration concerning the shear resistance of elements made with recycled aggregates produced from construction and demolition waste; and research in which the shear behaviour of concrete and the properties of the recycled aggregates are thoroughly characterised, so that the resistance models for shear design are appropriately changed with physically based coefficients rather than partial factors.

## Figures and Tables

**Table 1:** Cases of design for shear design of NAC slabs without shear reinforcement using prEN1992 [32].

Slab	$f_{ck}$ (MPa)	$d_{max}$ (mm)	H (mm)	c (mm)	L (m)	a/d	$\Phi_{Asl}$ (mm)	s (mm)	$\rho_{l}$	$V_{Rd}$ (kN)
1	25	20	170	25	5.0	2.5	12	100	0.81%	122.1
2	25	20	140	25	5.0	2.5	16	100	1.88%	135.5
3	25	20	210	25	5.0	2.5	16	150	0.76%	140.0
4	40	20	170	25	6.5	2.5	16	150	0.97%	150.4
5	40	20	145	25	6.5	2.5	16	75	2.39%	177.1

**Table 2:** Partial factors for elements without shear reinforcement used in the first iteration of the calibration procedures [24].

Code	$\gamma_{NAC}$	$\gamma_{RAC50}$	$\gamma_{RAC100}$
EN1992 [32]	1.45	1.55	1.60
prEN1992 [31]	1.40	1.60	1.70

**Table 3:** Cases of design for shear design of NAC slabs without shear reinforcement using EN1992 [31].

Slab	$f_{ck}$ (MPa)	H (mm)	c (mm)	L (m)	a/d	$\Phi_{Asl}$ (mm)	s (mm)	$\rho_{l}$	$V_{Rd}$ (kN)
1	25	190	25	5.0	2.5	16	104.1	1.23%	122.1
2	25	190	25	5.0	2.5	16	86.2	1.49%	135.5
3	25	215	25	5.0	2.5	16	100	1.10%	140.0
4	40	175	25	6.5	2.5	16	73	1.94%	150.4
5	40	200	25	6.5	2.5	20	94.2	2.02%	177.1

**Table 4:** Case of design for reliability analysis of shear design of NAC beam with shear reinforcement.

$f_{ck}$ (MPa)	b (mm)	H (mm)	c (mm)	d (mm)	$\Phi_{Asl}$ (mm)	$\Phi_{Asw}$ (mm)	s (mm)	$A_{sv}/s$ (cm^2^/mm)	$\rho_{w}$	$\rho_{w}$$\cdot f_{yk/\gamma_{S}}$ (MPa)	Ω	$V_{Rd}$ (kN)
25	250	450	25	407	16	8	150	6.7	0.27%	1.17	30°	185.9

**Table 5:** Deterministic and stochastic modelling of load-effects, geometry, and material properties.

Parameter	Deterministic Model		Stochastic Model				Reference
	Symbol	Fractile	Symbol	Mean	Standard Deviation (σ)	Probability Distribution	
Permanent load	$P_{k}$	50 %	P	$P_{k}$	0.10 × P	Normal	[44]
Maximum variable load (50 years)	$Q_{k}$	*	Q	0.60 $\times \;Q_{k}$	0.35 × Q	Gumbel	[65,66,67,68,69,70]
Model uncertainty of load-effects	Absent from deterministic modelling		$\theta_{E}$	1.00	0.05	Lognormal	[55,71]
Compressive strength	$f_{ck}$	5% fractile	$f_{c}$	$f_{c}=\lambda \times F_{2}\times f_{ck}$, with $f_{ck}$ assumed deterministic			[63,64]
Specified to delivered strength	Absent from deterministic modelling	λ	1.20	0.17			Lognormal
Standard to strength-in-structures	Absent from deterministic modelling	$F_{2}$	0.95	0.13			Lognormal
Yield stress of the reinforcement	$f_{yd}$	5% fractile	$f_{y}$	$f_{yd}+2\sigma$	30 MPa	Lognormal	[55,56,62,72];
Young’s modulus of the reinforcement	$E_{s}$ = 200 GPa assumed as deterministic						
Cross-sectional area of the reinforcement	$A_{s}$ assumed as deterministic						
Height of the cross-section	*H* assumed as deterministic						
Concrete cover (vertical)	cy	Nominal value	cy+Δcy	Δcy: 5 mm (slabs) Δcy: −5 mm (beams)	Δcy: 5 mm (slabs) Δcy: −5 mm (beams)	Δcy: Normal cy is deterministic	[13,62]

* $Q_{k}$ is conceptualized as the 98% fractile of the one-year maximum load, but this concept is not used for loads in buildings due to scarce statistical information [73]. The $Q_{k}$ of loads on buildings of EN1991 [74] are based on relevant reports on the subject and on engineering judgment [75,76,77].

**Table 6:** Stochastic modelling of the model uncertainty of elements without shear reinforcement.

$\theta_{R}$	Incorporation Ratio of RAs	Mean Value	Standard Deviation (σ)	Probability Distribution
EN 1992 [31]	NAC	1.03	0.113	Lognormal
	RAC50	1.00	0.120	Lognormal
	RAC100	0.95	0.114	Lognormal
prEN 1992 [32]	NAC	0.98	0.088	Lognormal
	RAC50	0.93	0.102	Lognormal
	RAC100	0.93	0.121	Lognormal

**Table 7:** Stochastic modelling of the model uncertainty of elements with shear reinforcement.

$\theta_{R}$	Incorporation Ratio of RA	Mean	Standard Deviation (σ)	Probability Distribution	Source
Assumption 1	NAC	1.25	0.312 (CoV = 25.0%)	Lognormal	NAC: *fib* Bulletin 80 [79] RAC: Same mean value as NAC; pessimistic expectation of the CoV
	RAC50	1.25	0.343 (CoV = 27.5%)	Lognormal	
	RAC100	1.25	0.375 (CoV = 30.0%)	Lognormal	
Assumption 2	NAC	1.25	0.312 (CoV = 25.0%)	Lognormal	NAC: *fib* Bulletin 80 [79] RAC: Pessimistic expectation of the mean value and CoV
	RAC50	1.21	0.333 (CoV = 27.5%)	Lognormal	
	RAC100	1.17	0.351 (CoV = 30.0%)	Lognormal	

**Table 8:** Ratio β RAC/β NAC of slabs. prEN1992 [32]. $\gamma_{NAC}=1.40$ and $\gamma_{CRAC50}=1.50;\;\gamma_{RAC100}=1.60$ (after calibration).

RA	Slab	*χ*						
		0%	10%	30%	50%	70%	90%	100%
RAC50	Slab 1	98%	98%	99%	101%	101%	102%	102%
	Slab 2	98%	98%	99%	101%	101%	102%	102%
	Slab 3	98%	98%	100%	101%	102%	102%	102%
	Slab 4	98%	98%	99%	101%	103%	103%	102%
	Slab 5	100%	102%	103%	104%	104%	104%	104%
RAC100	Slab 1	96%	96%	98%	100%	101%	102%	102%
	Slab 2	93%	92%	96%	99%	101%	102%	102%
	Slab 3	94%	93%	100%	97%	102%	102%	103%
	Slab 4	94%	93%	96%	100%	101%	102%	102%
	Slab 5	94%	93%	96%	99%	101%	102%	102%

**Table 9:** Ratio β RAC/β NAC of slabs. EN1992 [31]. $\gamma_{NAC}=1.45$; $\gamma_{RAC50}=1.55;\;\gamma_{RAC100}=1.60$ (no calibration needed).

RA	Slab	*χ*						
		0%	10%	30%	50%	70%	90%	100%
RAC50	Slab 1	100%	100%	100%	101%	101%	100%	102%
	Slab 2	100%	100%	100%	101%	101%	102%	102%
	Slab 3	102%	100%	101%	101%	101%	101%	102%
	Slab 4	100%	101%	100%	101%	101%	102%	102%
	Slab 5	101%	100%	100%	101%	101%	102%	102%
RAC100	Slab 1	98%	98%	99%	100%	100%	98%	100%
	Slab 2	98%	97%	98%	99%	100%	100%	100%
	Slab 3	101%	101%	98%	97%	101%	100%	99%
	Slab 4	98%	98%	99%	99%	100%	100%	100%
	Slab 5	98%	97%	98%	99%	100%	100%	100%

**Table 10:** Elasticities of $\theta_{VR,with\;shear}$ for *χ* = 0. EN1992 [31] and prEN1992 [32]. Assumption 1: $\gamma_{RAC50}=1.00;$ $\gamma_{RAC100}=1.05$; Assumption 2: $\gamma_{RAC50}=1.10;$ $\gamma_{RAC100}=1.20$ (after calibration).

Elasticity (%)	NAC	RAC50 Assumption 1	RAC100 Assumption 1	RAC50 Assumption 2	RAC100 Assumption 2
Mean	2.01	2.04	1.99	1.97	1.94
Standard deviation	−0.86	−0.88	−0.89	−0.89	−0.91

**Table 11:** Recommended partial factors for shear design of NAC and RAC elements.

	Design			
Type of Concrete	Shear without Shear Reinforcement		Shear with Shear Reinforcement—EN1992 [31] and prEN1992 [32]	
	EN1992 [31]	prEN1992 [32]	Assumption 1 (Moderate)	Assumption 2 (Pessimistic)
NAC	1.45	1.40	1.00	1.00
RAC50	1.55	1.50	1.00	1.10
RAC100	1.60	1.60	1.05	1.20

## Data Availability

The data presented in this study are available on reasonable request from the corresponding author.

## List of Acronyms and Symbols

CDW	construction and demolition waste
CoV	coefficient of variation
NA	coarse natural aggregate
RA	coarse recycled aggregate
RAC	concrete with partial or total incorporation of coarse recycled aggregate concrete
RAC50	recycled aggregate concrete elements with 50% incorporation of coarse recycled aggregates
RAC100	recycled aggregate concrete elements with full incorporation of coarse recycled aggregates
$A_{s}$	cross-sectional area of the reinforcement
$A_{sw}$	area of shear reinforcement
E	random outcome of load-effects
$E_{d}$	design value of load-effects
$E_{s}$	Young’s modulus of reinforcement
$F_{2}$	conversion of delivered strength measured on standard specimens to the strength within structural elements
H	height of the beam
$M_{cs}$	bending moment at the control section
P	random outcome of permanent loading
$P_{k}$	characteristic value of permanent loading
Q	random outcome of variable loading
$Q_{k}$	characteristic value of variable loading
R	random outcome of resistance
$V_{cs}$	shear stress at the control section
$V_{Rd}$	design value of shear resistance
$V_{Rd,cmin}$	design value of the minimum shear resistance of elements without shear reinforcement
$V_{Rd,strut}$	design value of the shear resistance of the compression struts of the resistance model of beams with shear reinforcement
$V_{Rd,tie}$	design value of the shear resistance of the ties of the resistance model of beams with shear reinforcement
b	width of the web of the beam
c	concrete cover
d	effective depth of the beam
$d_{max}$	maximum aggregate diameter
$f_{c}$	random outcome of the compressive strength of concrete
$f_{ck}$	characteristic value of the compressive strength of concrete
$f_{y}$	random outcome of the yield stress of the reinforcement
$f_{yd}$	design value of the yield stress of the reinforcement
$f_{yk}$	characteristic yield stress of the reinforcement
$g_{x}$	limit state function
s	distance between reinforcement
ΔB	uncertainty in the width of the beam
$\Phi_{Asl}$	diameter of longitudinal reinforcement
Ω	angle of the compression strut with the longitudinal axis used in the resistance model of elements with shear reinforcement
*α*	direction cosine of a stochastic variable
*β*	reliability index
$\beta_{target}$	target reliability index
$\gamma_{C}$	partial factor for the strength of concrete
$\gamma_{c}$	partial factor for the variability of the strength of concrete
$\gamma_{G}$	partial factor for permanent loads
$\gamma_{Q}$	partial factor for variable loads
$\gamma_{NAC}$	partial factor for shear design of natural aggregate concrete elements without shear reinforcement
$\gamma_{RAC}$	partial factor for shear design of recycled aggregate concrete elements
$\gamma_{RAC}$	partial factor for shear design of recycled aggregate concrete elements with 50% incorporation of coarse recycled aggregates
$\gamma_{RAC100}$	partial factor for shear design of recycled aggregate concrete elements with full incorporation of coarse recycled aggregates
$\gamma_{Rd}$	partial factor for the uncertainty in geometry and in resistance modelling
$\gamma_{S}$	partial factor for the yield stress of the reinforcement
$\theta_{E}$	model uncertainty of load-effect modelling
$\theta_{R}$	model uncertainty of the resistance model
λ	stochastic model for the conversion of specified to delivered strength
$\rho_{l}$	geometric ratio of longitudinal tensile reinforcement
$\rho_{w}$	geometric ratio of shear reinforcement
$\nu_{min}$	minimum shear stress of the resistance model of elements without shear reinforcement
χ	ratio of the design value of the variable loading to the total design value of loading
